# Successful surgical repair of aortic root rupture during transcatheter aortic valve implantation: a case report

**DOI:** 10.1186/s44215-024-00172-4

**Published:** 2024-10-28

**Authors:** Ryosuke Numaguchi, Noriyuki Yokoyama, Kazunori Ishikawa, Atsuhiro Koya, Yusuke Tokuda, Masami Shingaki

**Affiliations:** 1 Department of Cardiovascular Surgery, Hakodate Municipal Hospital, 1-10-1 Minato-cho, Hakodate City, Hokkaido 041-8680 Japan; 2 Department of Cardiology, Hakodate Municipal Hospital, 1-10-1 Minato-cho, Hakodate City, Hokkaido 041-8680 Japan

**Keywords:** Transcatheter aortic valve implantation, Aortic valve replacement, Aortic root rupture, Aortic root repair

## Abstract

**Background:**

Aortic root rupture is a rare but potentially fatal complication of transcatheter aortic valve implantation (TAVI). Herein, we report a case of aortic root rupture during TAVI that was successfully managed with partial aortic root repair and aortic valve replacement.

**Case presentation:**

An 83-year-old woman with severe bicuspid aortic stenosis underwent transfemoral TAVI using a 26 mm SAPIEN 3 Ultra RESILIA valve. Soon after valve implantation, transesophageal echocardiography detected pericardial effusion, which was accompanied by immediate hemodynamic instability. Subxiphoid pericardial drainage was performed, and extracorporeal membrane oxygenation was promptly initiated. Owing to the eruptive hemorrhage, we converted to open-heart surgery. Direct visualization enabled the detection of the aortic root rupture and the performance of partial aortic root repair using bovine pericardium and aortic valve replacement using a 19 mm INSPIRIS RESILIA valve. The patient was transferred to a different hospital 60 days after surgery without complications and is currently doing well as an outpatient.

**Conclusions:**

In the present case, prompt treatment decisions and smooth cooperation among the institutional heart team led to the successful repair of aortic root rupture during TAVI and good clinical outcomes.

## Background

Aortic root rupture is a rare but life-threatening complication of transcatheter aortic valve implantation (TAVI). The clinical severity and management of aortic root rupture depend on the location and extent of the injury [1]. If rapid hemodynamic deterioration occurs, prompt decision-making and smooth coordination among the heart team are vital to save the life of the patient.

Here, we describe a case of aortic root rupture during TAVI that was successfully managed with partial root repair and aortic valve replacement (AVR). This case report was approved by the Hakodate Municipal Hospital Research Ethics Committee (approval no. 2023–306).

## Case presentation

**Fig. 1:**
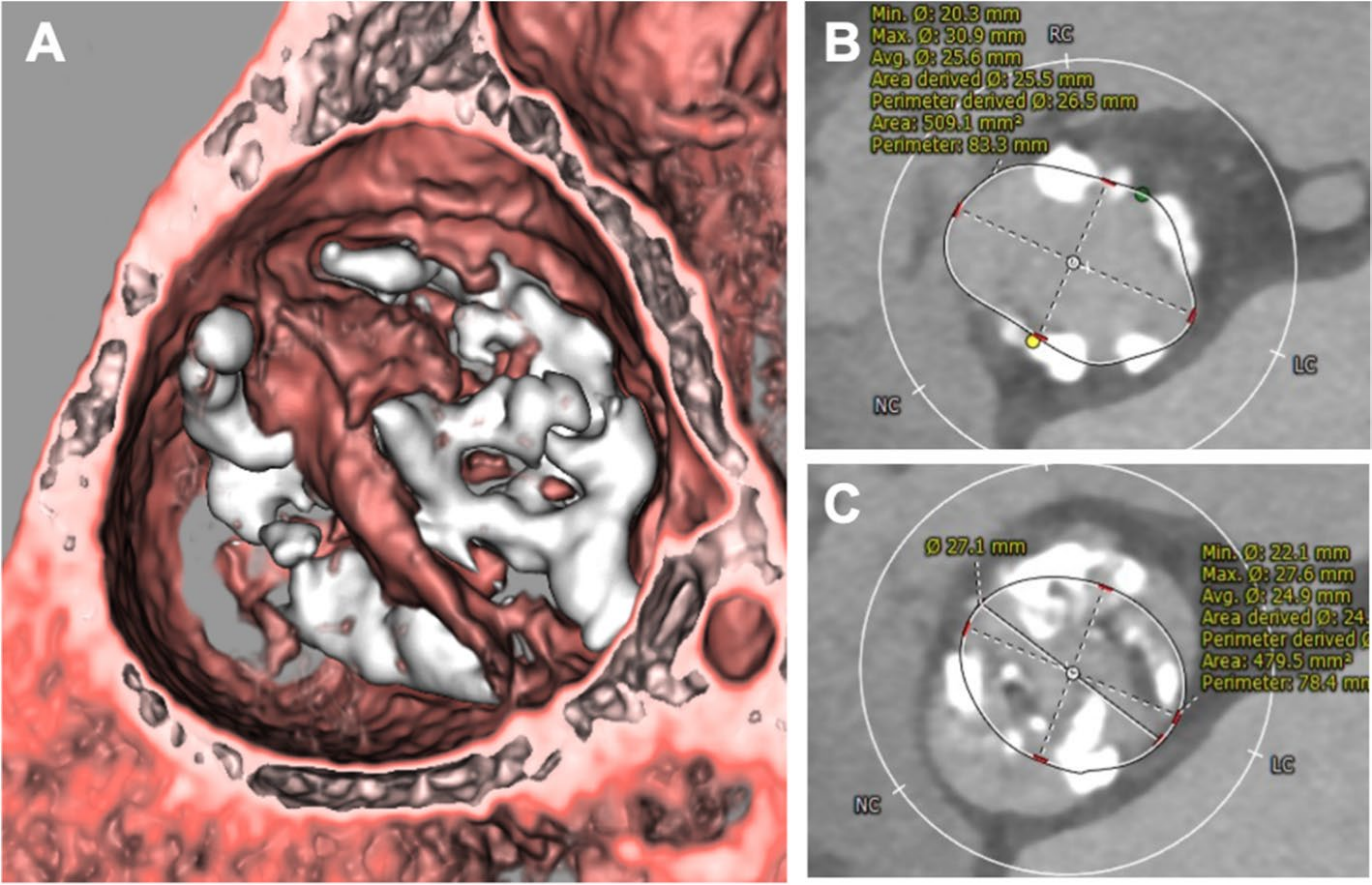
**A** Computed tomography scan shows severely calcified bicuspid aortic valve. **B** Measurements of the annular plane indicated an annular area of 509 mm^2^ and an annular perimeter of 83.3 mm. **C** Measurements of the leaflet plane. A 26 mm SAPIEN3 valve was expected to be positioned as shown in the figure

**Fig. 2:**
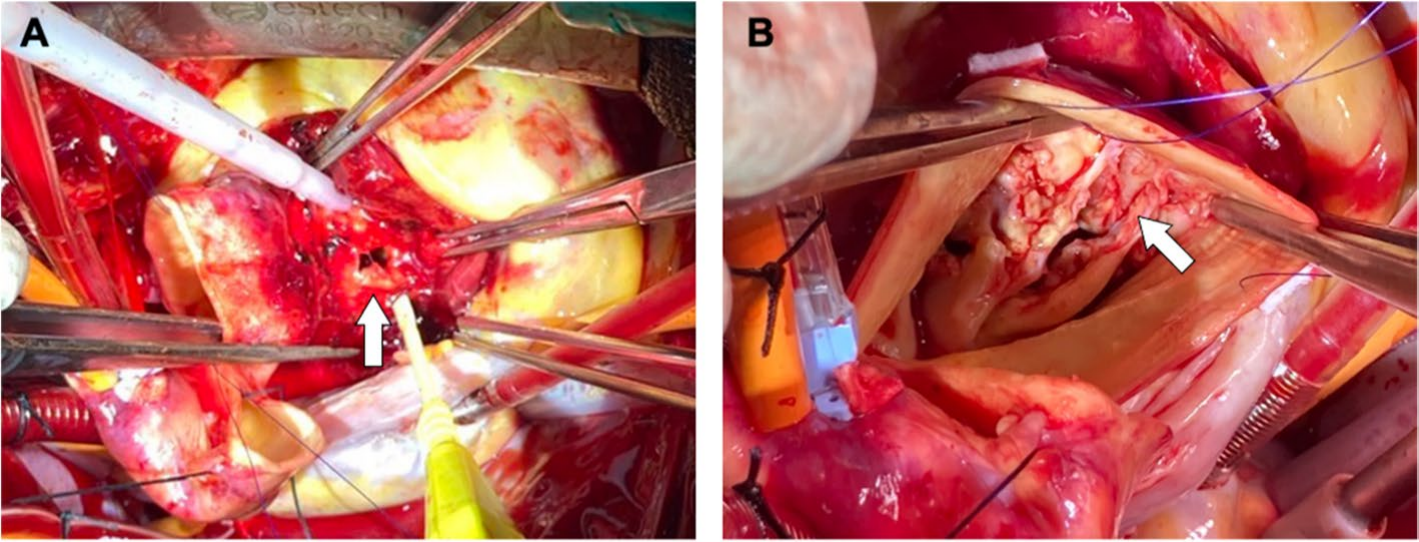
Intraoperative findings of the anterior portion of the sinus of Valsalva. **A** A 5 mm tear is observed from the outside of the sinus of Valsalva (white arrow). **B** The anterior commissure was torn due to the dilation of the implanted valve (white arrow), resulting in aortic root rupture

**Fig. 3:**
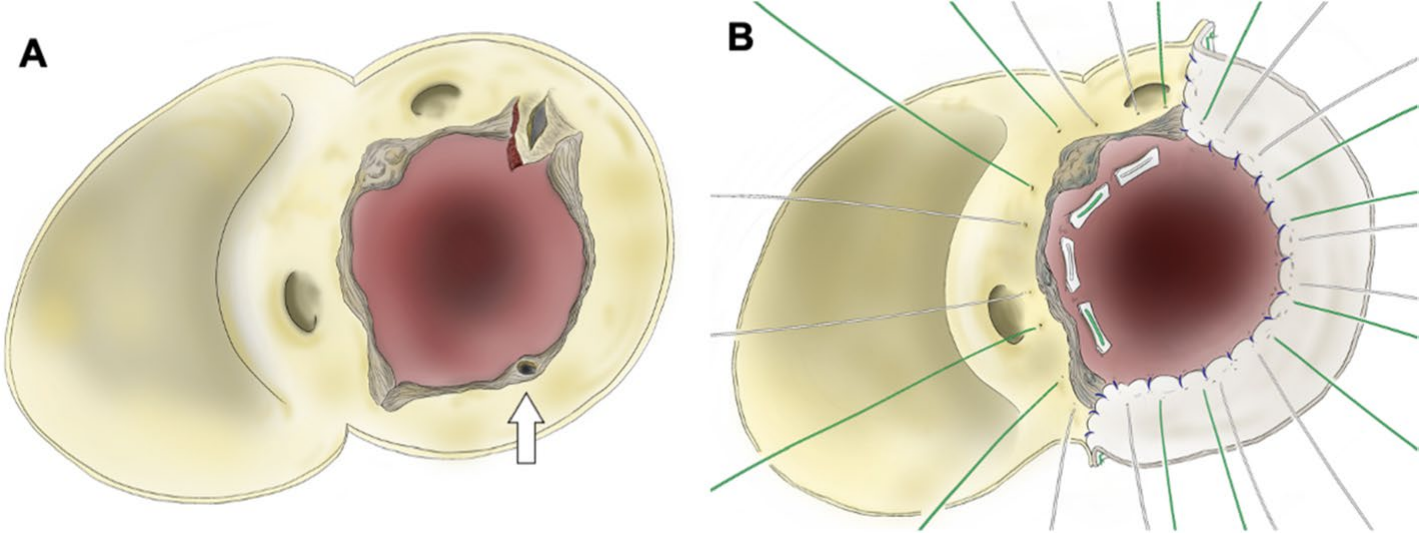
Schematic illustrations of aortic root repair. **A** After removing the native aortic valve, a part of the annulus was unintentionally damaged due to aggressive debridement (white arrow). **B** The injured annulus was reconstructed using bovine pericardium, and a new valve was subsequently positioned and secured with sutures placed in a non-everting fashion in the native aortic annulus and in a transmural outside-in fashion in the patch region

An 83-year-old woman (height, 1.55 m; weight, 31.9 kg; body surface area, 1.2 m^2^) was referred to our hospital with symptomatic severe aortic stenosis. Her medical history included hypertension, osteoporosis, and microscopic polyangiitis. She was receiving monthly intravenous cyclophosphamide pulse therapy and 20 mg of oral prednisolone. Transthoracic echocardiography revealed a heavily calcified bicuspid aortic valve with fusion of the right and left coronary cusps, an aortic valve area of 0.45 cm^2^, and a peak velocity of 5.1 m/s. Computed tomography revealed an annular plane area of 509 mm^2^ with nodular calcifications in the left ventricular outflow tract (LVOT) (Fig. 1). Severely calcified bicuspid valve leaflets and nodular calcifications in the LVOT are risk factors for TAVI-related complications. However, conventional surgical AVR was not considered feasible in this patient because of her advanced age, moderate surgical risk (STS operative mortality score: 5.54%), and frailty (Clinical Frailty Scale [2]: 5). Regarding the device selection, the use of self-expandable valve may help mitigate the risk of annular rupture. However, significant calcification could potentially distort the valve opening, leading to insufficient function and unacceptable aortic valve regurgitation. We anticipated that the use of an underfilled balloon dilatation could avoid the risk of annular rupture due to the use of a balloon-expandable valve. Ultimately, we planned to perform transfemoral TAVI using a 26 mm SAPIEN 3 Ultra RESILIA valve (Edwards Lifesciences, Irvine, CA, USA) with a 1 mL underfilled dilation. During the procedure, the valve was implanted using slow and careful dilation to minimize the risk of aortic root complications. However, soon after valve implantation, transesophageal echocardiography revealed pericardial effusion, and the hemodynamic status of the patient deteriorated rapidly. Prompt pericardial drainage was performed via a subxiphoid skin incision and femoro-femoral extracorporeal membrane oxygenation (ECMO) was initiated within 10 min of effusion detection. The hemorrhage was uncontrollable, and we therefore converted to open-heart surgery. A median sternotomy was performed, allowing the detection of a pulsatile hemorrhage in the anterior region of the sinus of Valsalva, indicating aortic rupture. Under aortic cross-clamping, the ascending aorta was opened, and a meticulous inspection of the aortic root was conducted. The valve had been implanted in the anterior commissural side of the annulus, and mechanical stress from the expanding valve had torn the anterior commissure. The valve was easily removed using two peans compressing and deforming the stent cage. After valve explantation, a 5 mm tear was observed just below the anterior commissure (Fig. 2). After removing the native valve leaflets, the severely damaged Valsalva sinus wall and aortic annulus were resected, leaving the bilateral coronary ostia intact. The resected area was reconstructed using a bovine pericardium patch with a 2–0 pledgeted mattress suture and 4–0 polypropylene running suture (Fig. 3). A 19 mm INSPIRIS RESILIA aortic valve (Edwards Lifesciences) was then implanted in the supra-annular position with 2–0 pledgeted valve sutures placed in a non-everting fashion in the native aortic annulus and a transmural outside-in fashion in the patch region (Fig. 3B). The patient was weaned smoothly from cardiopulmonary bypass. Aortic cross-clamp and cardiopulmonary bypass times were 135 and 190 min, respectively. Although the patient experienced some postoperative rehabilitation difficulties, she was successfully transferred to another hospital 60 days after surgery without complications and is currently doing well as an outpatient.

## Discussion

In this report, we describe a case of aortic root rupture during TAVI that was successfully managed with emergency open-heart surgery. Aortic root rupture is a rare complication of TAVI, with a reported incidence of 0.13–1.0% [3–5]. Management depends on the location of the rupture and its clinical manifestations. In less severe cases, a conservative approach or isolated pericardial drainage can be effective [6–8]. However, hemodynamic status can often deteriorate rapidly, necessitating urgent initiation of ECMO and conventional open-heart surgery. As the type of surgical procedure required varies depending on the location and severity of the rupture, an ad hoc surgical strategy is needed. In the present case, the primary injury was located in the anterior commissure, and the intact aortic annulus around the coronary ostia indicated that aortic root replacement could be avoided. As a technical point of reflection, the non-coronary sinus annulus was unintentionally damaged during native valve resection, necessitating a patch repair of the region. It is speculated that balloon-expandable valve deployment can cause invisible tears in the native aortic annulus. Therefore, complete decalcification, as typically performed in typical AVR, could lead to secondary annular injury. Consequently, annular debridement should be minimized to simplify the procedure.

In cases of suspected annular rupture accompanied by rapid hemodynamic deterioration, the primary therapeutic goal is to achieve hemodynamic stability promptly by relieving cardiac tamponade and initiating extracorporeal circulation. In such cases, the priority is to initiate ECMO via peripheral access, while subsequent treatment should be carefully determined after ensuring stable circulation. As part of emergency preparedness, stand-by ECMO is typically set up in the operating room, and dedicated femoral sheaths are inserted via the femoral artery and vein to facilitate emergent ECMO initiation in all cases, enabling extremely rapid ECMO initiation.

In this case, we performed TAVI with a 26 mm SAPIEN3 Ultra RESILIA valve for a measured annular plane of 509 mm^2^, in accordance with the recommended sizing guidelines. Unfortunately, an annular rupture occurred; however, given that the patient was over 80 years old and had a moderate surgical risk, with an STS mortality score of 5.54%, the indication for TAVI itself was appropriate. However, alternative strategies should have been more carefully considered because the patient had a significant risk of annular rupture. First, the use of a self-expandable valve should provide a safe solution in cases of severely calcified valve leaflets and nodular calcification in the LVOT, even if there is a risk of paravalvular leakage. Second, using a downsized valve or further underfilling the balloon may be a feasible strategy in patients at risk of aortic root complications, because SAPIEN3 Ultra valves have a greater inhibitory effect on paravalvular leakage owing to the improved sealing skirt [9].

## Conclusion

In cases of aortic root rupture accompanied by rapid hemodynamic instability during TAVI, the priority is to achieve stable hemodynamic circulation through prompt treatment decisions and smooth coordination among the heart team. Although aortic root rupture is a life-threatening complication, adequate preparedness and treatment decisions may lead to good outcomes.

## Data Availability

Data sharing is not applicable to this article as no datasets were generated or analyzed during the current study.

## Abbreviations

TAVI: Transcatheter aortic valve implantation
AVR: Aortic valve replacement
LVOT: Left ventricular outflow tract
ECMO: Extracorporeal membrane oxygenation
